# Prostate cancer proteomics: Current trends and future perspectives for biomarker discovery

**DOI:** 10.18632/oncotarget.14501

**Published:** 2017-01-04

**Authors:** Cristiana Pistol Tanase, Elena Codrici, Ionela Daniela Popescu, Simona Mihai, Ana-Maria Enciu, Laura Georgiana Necula, Adrian Preda, Gener Ismail, Radu Albulescu

**Affiliations:** ^1^ Department of Biochemistry-Proteomics, Victor Babes National Institute of Pathology, Bucharest, Romania; ^2^ Titu Maiorescu University, Faculty of Medicine, Bucharest, Romania; ^3^ Department of Cell Biology and Histology, Carol Davila University of Medicine and Pharmacy, Bucharest, Romania; ^4^ Stefan S Nicolau Institute of Virology, Bucharest, Romania; ^5^ Center for Uronephrology and Renal Transplantation, Fundeni Clinical Institute, Bucharest, Romania; ^6^ Center of Internal Medicine-Nephrology, Fundeni Clinical Institute, Bucharest, Romania; ^7^ National Institute for Chemical Pharmaceutical R&D, Bucharest, Romania; ^8^ Carol Davila University of Medicine and Pharmacy, Faculty of Medicine, Bucharest, Romania

**Keywords:** prostate cancer, biomarkers, proteomics, mass spectrometry, prostasomes

## Abstract

The clinical and fundamental research in prostate cancer - the most common urological cancer in men - is currently entering the proteomic and genomic era. The focus has switched from one single marker (PSA) to panels of biomarkers (including proteins involved in ribosomal function and heat shock proteins). Novel genetic markers (such as Transmembrane protease serine 2 (TMPRSS2)-ERG fusion gene mRNA) or prostate cancer gene 3 (PCA3) had already entered the clinical practice, raising the question whether subsequent protein changes impact the evolution of the disease and the response to treatment. Proteomic technologies such as MALDI-MS, SELDI-MS, i-TRAQ allow a qualitative/quantitative analysis of the proteome variations, in both serum and tumor tissue. A new trend in prostate cancer research is proteomic analysis of prostasomes (prostate-specific exosomes), for the discovery of new biomarkers. This paper provides an update of novel clinical tests used in research and clinical diagnostic, as well as of potential tissue or fluid biomarkers provided by extensive proteomic research data.

## INTRODUCTION

Prostate cancer (PCa) is the leading type of most common diagnosed urological cancer in men, and its prevalence is continuously increasing. Furthermore, PCa is currently the second leading cause of cancer-specific death in many countries [1]. It is usually diagnosed on the basis of digital rectal examination (DRE), prostate-specific antigen (PSA) serum levels and multicore schemes of prostate biopsy. Notably, PCa is a very heterogeneous disease characterized by different clinical behavior, from indolent to aggressive tumors with lethal progression. Therefore, early diagnostics and identification of PCa aggressiveness are crucial prerequisites for efficient treatment of patients [2]. Efforts are made continuously by researchers to investigate new potential biomarkers for a better risk stratification and personalized treatment strategy, given its variability in clinical behavior, treatment decisions and therapeutic responses [3]. An important advancement in proteomics is the quantification of biomarkers, provided by new and powerful platforms, from both fluids (urine, blood, seminal fluid) and tissue [4]. This paper provides an overview of current most promising biomarkers identified with the new *-omics technologies* to help physicians in clinical decision making for PCa diagnosis, prognosis and prediction of therapeutic effect.

## NEW CLINICAL BIOMARKERS FOR PCA DIAGNOSIS, RISK STRATIFICATION AND AGGRESSIVENESS

The discovery of PSA as a serum marker has revolutionized PCa diagnosis and nowadays is the only widely used PCa biomarker for diagnosis and prognosis of this disease. However, PSA is organ- but not cancer specific. Moreover, it is not able to differentiate between indolent and aggressive PCa. In addition, many men may harbor aggressive PCa disease despite having low initial value of serum PSA [5]. However, total PSA serum value together with Gleason score are the most significant variables to identify men at increased risk of PCa and are included in all nomograms for an accurate risk stratification of patients with PCa, both at the time of diagnosis and post-treatment [6].

Establishing the PCa aggressiveness and the optimal moment for therapeutic intervention are the primary end-points of the current clinical trials that are trying to identify new potential biomarkers for a better insight into PCa natural history [7].

In the era of personalized medicine, a number of novel biomarkers become available to guide physicians in difficult clinical-decision making.

A promising biomarker for PCa diagnosis is prostate cancer gene 3 (PCA3) which is highly over-expressed by prostatic cancer cells. This prostate-specific gene is a non-coding mRNA biomarker that can be found in urine specimens collected after DRE. An *in vitro* nucleic acid amplification test called Progensa™ PCA3 test was developed by Gen-Probe Inc. (San Diego, CA, USA) and is now commercially available for the use in patients with previous negative biopsy results for whom a repeat biopsy is considered by an urologist based on PSA level or DRE to predict positive biopsies (malignancy). The assay calculates the ratio of PCA3 mRNA levels to PSA mRNA levels to generate PCA3 score. A score of less than 25 indicates a decrease probability of a positive repeat biopsy. This test was shown to be superior to total and free PSA for PCa diagnosis as demonstrated by numerous validation studies. For example, data from Deras et al. indicate that PCA3 diagnostic accuracy was greater than PSA as demonstrated by AUC (0.703 *vs*. 0.618) [8]. On the contrary, clinical utility and superiority of PCA3 was not demonstrated in patients undergoing first biopsy settings [9]. Also, it cannot distinguish between high grade PIN (Prostatic Intraepithelial Neoplasia) and PCa [10] and its utility as a prognostic test for PCa aggressiveness remain to be investigated. However, recent data indicate that PCA3 has a promising role for monitoring in active surveillance since it can differentiate between high grade PIN and low-volume PCa [11].

Researchers tried to improve the risk assessment of PCa by combining the urine tests for PCA3 from Progensa with T2:ERG, and serum PSA levels and developed Mi-Prostate Score [12]. This test is offered by Mlabs, University of Michigan and was validated by numerous studies [13]. AUC was significantly greater than models incorporating PCA3 and PSA alone for the prediction of PCa or high grade disease on biopsy and provided an increase of sensitivity and specificity to 80% and 90% respectively [12]. Transmembrane protease serine 2 (TMPRSS2)-ERG fusion gene is one of the most common genomic alterations identified in about 50% of prostate cancer (urine or tissue). Although, TMPRSS2-ERG overexpression has high prostate cancer specificity, its role in detecting aggressive prostate cancer is still controversial. A recent report of a prospective multicentric trial concluded that TMPRSS2-ERG had also higher predictive value unlike PCA3 score [14].

PROSTARIX™ is another commercially available urinary test developed by Metabolon Inc. (Durham, NC, USA) in agreement with Bostwick Laboratories (Glen Allen, VA, USA). This non-invasive urinary test measures a panel of four metabolites (alanine, glutamate, glycine, sarcosine) by chromatography and mass spectrometry after a vigorous DRE. This product was developed to help clinicians in their decision of PCa detection on the first or subsequent set of prostate biopsy in patients with PSA level between 2-15 ng/ml and negative or suspicious DRE [15]. The Prostarix test showed increased sensitivity and specificity over serum PSA and its diagnostic accuracy was further improved by addition of clinical findings into a logistic regression model (AUC 0.78).

ConfirmMDx (MDx Health, Irvine, CA) is an epigenetic test that uses normal or benign prostate cores specimens for the prediction of a positive subsequent prostate biopsy to help patients to make adequate informed consent about the management of PCa at the initial diagnosis [16]. Its clinical utility to reduce the need for rebiopsy and detect latent disease was proven in several trial studies such as Matloc study or Document Study [13, 16]. Indeed, this test had a sensitivity and specificity of 62-68 and 64 % respectively with a negative predictive value of 88-90%.

Other tests that can reduce unnecessary prostate biopsies and more important to identify aggressive disease (Gleason score > 7 or extraprostatic extension) are ProMark (tissue based) and 4K Score (blood based). ProMark^®^ (Metamark Genetics, Inc, Cambridge, MA) is a tissue test with prognostic value for PCa aggressiveness; detects a 8-proteins signature (DERL1, CUL2, SMAD4, PDSS2, HSPA9, FUS, pS6 (phosphorylated S6), YBOX1), not influenced by sampling error [17]. 4Kscore^®^ Test (OPKO Lab, Nashville, TN) is a promising blood test not yet FDA-approved that measures Total PSA, Free PSA, Intact PSA, and Human Kallikrein 2 (hK2) to establish the probability of detecting an aggressive (Gleason score 7 or higher) PCa upon biopsy. An algorithm is generated by combining the blood test results with patient parameters (age, DRE and previous biopsy results). For both scores, the validation studies demonstrated the improvement of PCa diagnosis and facilitate clinical decisions for localized PCa, stratifying patients for active surveillance or therapeutic interventions [18].

Oncotype DX^®^ test (Genomic Health, Inc,, Redwood City, CA) was developed as a biopsy-based genomic assay to predict adverse pathology and to distinguish between indolent and aggressive disease [19]. The test is a RT-PCR expression array of 12 genes implicated in PCa tumorigenesis (angiogenesis, proliferation, cellular organization and stromal response) and uses small (1 mm) fixed paraffin-embedded tissue samples from needle biopsies. The Genomic Prostate Score [20] (0-100, higher scores indicate more aggressive disease) was externally validated as a significant predictor of aggressive disease and helps clinicians to identify high risk patients and start immediate adapted therapy.

Another test clinically validated in multiple cohorts to identify aggressive disease is Prolaris^®^ (Myriad Genetic Laboratories inc, Salt Lake City, UT). This assay measures the expression of 31 cell cycle progression (CCP) genes selected because of their demonstrated correlation with PCa proliferation, against 15 housekeeper genes from formalin-fixed paraffin-embedded tissue obtained by prostate biopsy or radical prostatectomy. The value of CCP test as a significant predictor for risk assessment beyond conventional clinic-pathological criteria on prostate biopsies and disease progression, recurrence, or prostate-cancer specific mortality on radical prostatectomy specimens was demonstrated in multiple cohorts [21]. Thus, it is an important tool for assessment of prognosis that helps clinicians to counsel their patients about how aggressive is disease prognosis and the need for close monitoring or adjuvant therapy [22].

Circulating Tumor Cells (CTC) Test for Prostate Cancer (CELLSEARCH^®^ Janssen Diagnostics, LLC, US) is FDA approved only for monitoring prostate cancer patients with metastatic disease and is not suitable for monitoring prostate cancer patients with non-metastatic disease [23].

## PROTEOMICS DISCOVERY PLATFORMS USED IN PROSTATE CANCER BIOMARKERS

In current *-omics* era, discovery and validation of protein biomarkers are essential for both research and clinical practice having huge impact on early cancer detection, diagnosis improvement, recurrence prevention, therapeutic response monitoring and increased survival outcome. Developing cancer risk-identifier biomarkers that aid both early detection and targeted therapy constitutes an essential aim of the oncology field.

Innovative high-throughput proteomic platforms are now available to generate complex protein profiles, representing an important concern in cancer research and clinical application. In this regard, clinical proteomics aims to identify and quantify new specific and sensitive biomarkers for PCa early detection, patient stratification and treatment efficacy. Many of these biomarkers were still need rigorous validation for being applied in clinical practice.

In the last decades, a major progress was recorded on identification of thousands of proteins, as candidate biomarkers, in complex biological systems by newly proteomic approaches - mass spectrometry, 2D electrophoresis, multiplex assays and protein microarrays [24].

The utilization of proteomic signatures based on circulating biomarker panels represents an encouraging approach for efficient monitoring of disease progression, as well as therapy [25, 26].

The “core” of almost all platforms is a “preliminary” sorting of molecules of interest, that can be achieved *via* several separation technologies, such as electrophoretic (most applied being 2D electrophoresis and its upgraded version 2D-DIGE). 2D/DIGE represents a “common” approach in the discovery of PCa biomarkers, using as starting material serum, plasma, tissue samples from patients, as well as various cell cultures featuring the cancers under observation [27]. Using the 2D-DIGE approach, 118 proteins with significantly altered expression were identified by Ummanni et al. in prostatic tissue tumor *vs*. peritumoral extracts [28]; another similar study by Davalieva et. al. [29] outlined 38 such spots, but in a more narrow pH interval, while Geisler et. al. [30] reports a set of 35 dysregulated proteins in prostatic tumor tissue. However, this technology needs the help of other proteomics instruments, such as MALDI-MS [29]. Similarly, proteins separated by 2D-DIGE were further analyzed after digestion of proteins in differentially expressed spots *via* the use of MALDI-TOF/MS-MS and LC/MS-MS [31].

While MALDI platforms are the most frequent MS instruments for resolving the proteomic composition, another similar platform, SELDI (Matrix Assisted Laser Desorption Ionization) was also used in proteomics [32]; a major distinct feature of SELDI is the resolution of integral proteins, and not of peptide fragments. Such studies were applied for serum samples [33, 34] or on urine samples [35].

Using isobaric tags for relative and absolute quantitation labeling (iTRAQ) and two dimensional-liquid chromatography-tandem MS, led to the identification of proteins with different glycosylation sites in cell lines of prostate cancer [36].

Apart for the laser ionization platform (SELDI, MALDI), other MS based platforms are present in proteomics: triple quadrupole mass spectrometers are the most commonly found in laboratories and used for quantitative analysis. The “triple quadrupole” is a cascade of quads, the first performs m/z sorting, the second fragments ions from the first filtering stage, while the third quad selects specific fragmentation products generated [37] .

SRM-MS (Single Reaction Monitoring) was used for discovery and validation of cancer biomarkers in serum [38], including PCa biomarkers [39, 40], as well as the MRM-MS approach (Multiple Reaction Monitoring - MS [37].

Mass spectrometry is used widely both in PCa and in other urological malignancies, in order to identify new biomarkers in tissue, blood and urine [41]. Therefore, it is a very promising technique in terms of implementation as a noninvasive clinical diagnosis tool.

### Analytical and clinical validation

Analytical validation: overall, concerns issues of the analytical procedures, and will regard assessments of intra- and inter-assay variability [42]. The target is thus on the analytical stages, which can be based on single instrumental approaches (SELDI-TOF, MALDI-TOFs), or on multiple instruments (like the combinations of LC-MS, CE-MS, 2DE-MS, etc.). There are some variants of assessment, depending on the methods or set of methods to be used in the process. Most often, the biomarker “discovery” stage is performed using high performance equipment (LC-MS, LS-MS/MS, MALDI TOF or MALDI TOF/TOF), rendering a molecular signature based on “molecular weights”, followed by identification in data bases or by more sophisticated fragmentation techniques. Several approaches can be exemplified for the validation procedures. From the discovery stage further, is possible to transfer to more simplified instrumentation, such as ELISA or multiple ELISAs, multiplex assays and microarrays.

A first step, which can be considered validation of the analytical stage in MS approaches, usually employs “synthetic” mixtures of proteins or peptides (depending on the specific instruments used, in order to perform the calibration and then the qualification of the equipment. The use of the same protein sets can be used at later stages for the purpose of calibrating the equipment in multi-site studies. An interesting example is offered in [43], where multiple peptides were analyzed in a multi-centric study. Although this study does not aim to identify prostate cancer biomarkers prostate cancer biomarkers, it is, until now, the best documented example of multi-platform validation, contributing to the improvement of targeted proteomics analyses.

Another approach uses “cross-method” approaches, after discovery using one technology, the “candidate” biomarker being validated by a second or third method of detection/quantitation. An example is provided in a study focused on the identification of metastatic progression biomarkers in PCa [44]. In this case, the discovery relied on iTRAQ, while the validation step relied on electrophoresis (1D), western-blot and IHC, and for some markers, by PCR.

A larger number of studies that use MALDI or SELDI TOF are in the “analytical” stage, based generally on the use of “synthetic mixtures” of proteins or peptides, that serve in generating calibration equations to be further used in establishing the mass-signatures of the samples. These synthetic mixtures are recommended in periodical calibration of the MS devices, as well as in multiplatform/ multi-center studies. Such protocols are available and applied on almost all equipment, and are readily obtainable from the suppliers.

A second stage, more related to clinical validation, involves several phases. In the development of such a protocol of identification of biomarkers, there is initially a “non-validation” stage, often named “training” - consisting in the acquisition of proteome data, followed by the selection of the marker/markers used in classification of the condition to be discriminated (diagnostics, prognostics etc). This step is achieved on the basis of “labeled” samples, assigned based on other methods, to the groups “patients” (eventually differentiated in subgroups, depending on disease stage, severity etc.) and “control”. Selection of markers generates then a “diagnostic algorithm”, and even in this stage some performance characteristics can be established (for instance, sensitivity, specificity, true positives and false positives, true and false negatives), or, for each of the selected markers, also the occurrence in the analyzed population.

The effective validation involves several stages, as follows:

Pre-validation - that can be achieved on some already run samples, (but in anonymized status - no tags for patient or control. The discriminator(s) are the identified biomarker(s), and based on theirs specific values, the results are sorted out in two groups, patients (or, for instance “cured”, ameliorated, etc.) and controls. There will presumably be some bias of the results, with some possible misclassifications (patients to controls and vice-versa). A second validation stage is often achieved on larger data sets (possible run in the same laboratory or in multiple laboratories. This turn is achieved mostly on new samples, completely anonymized, however, in some techniques is often applied the run of reference samples (such as a mixture of samples from several patients and/or patients and controls), the reason being that by this approach the broadest proteomic diversity is repeatedly run, providing an internal standard for inter-assay validation. Some such examples are illustrated for instance, for the applications of SELDI and MALDI techniques in proteomics [45–48].

**Figure 1:**
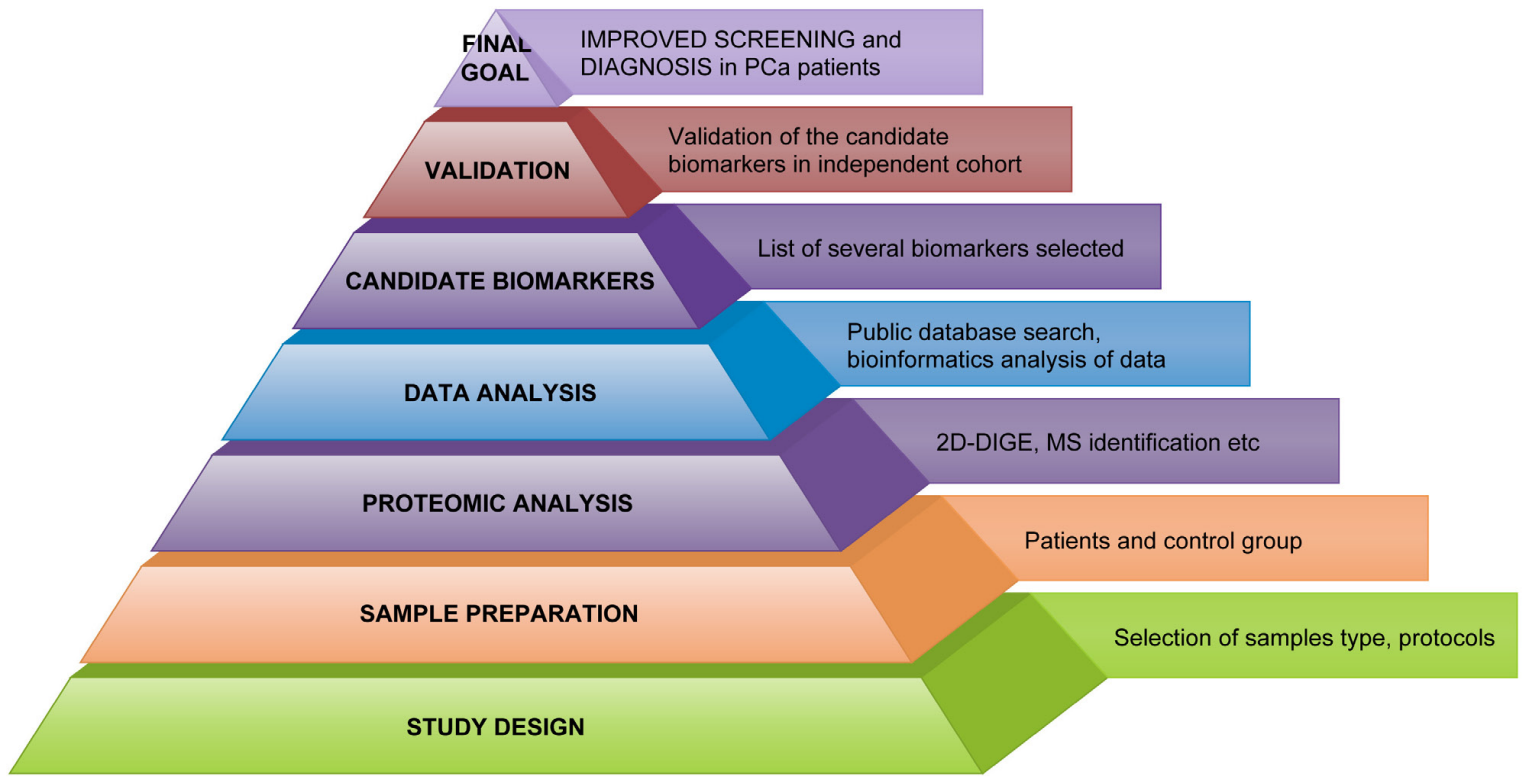
Workflow in Workflow in PCa proteomics

In this respect, high-throughput proteomic technologies will hold a greater value in PCa approaches/clinical management, as a basic step in improved diagnostic and prognostic (Figure 1).

## PROTEOMIC BIOMARKERS FOR PROSTATE CANCER

Cancer biomarkers are usually classified into three categories: prognostic, predictive, and pharmacodynamics [25]. *Prognostic* biomarkers predict the natural course of cancer and distinguish the tumor's outcome. They also help determine whom to treat, how aggressively to treat, and which candidates will likely respond to a given drug and the most effective dose. *Predictive* biomarkers evaluate the probable benefit of a particular treatment. *Pharmacodynamics* biomarkers assess the imminent treatment effects of a drug on a tumor and can possibly determine the proper dosage in the early stages of clinical development of a new anticancer drug [49, 50].

**Figure 2:**
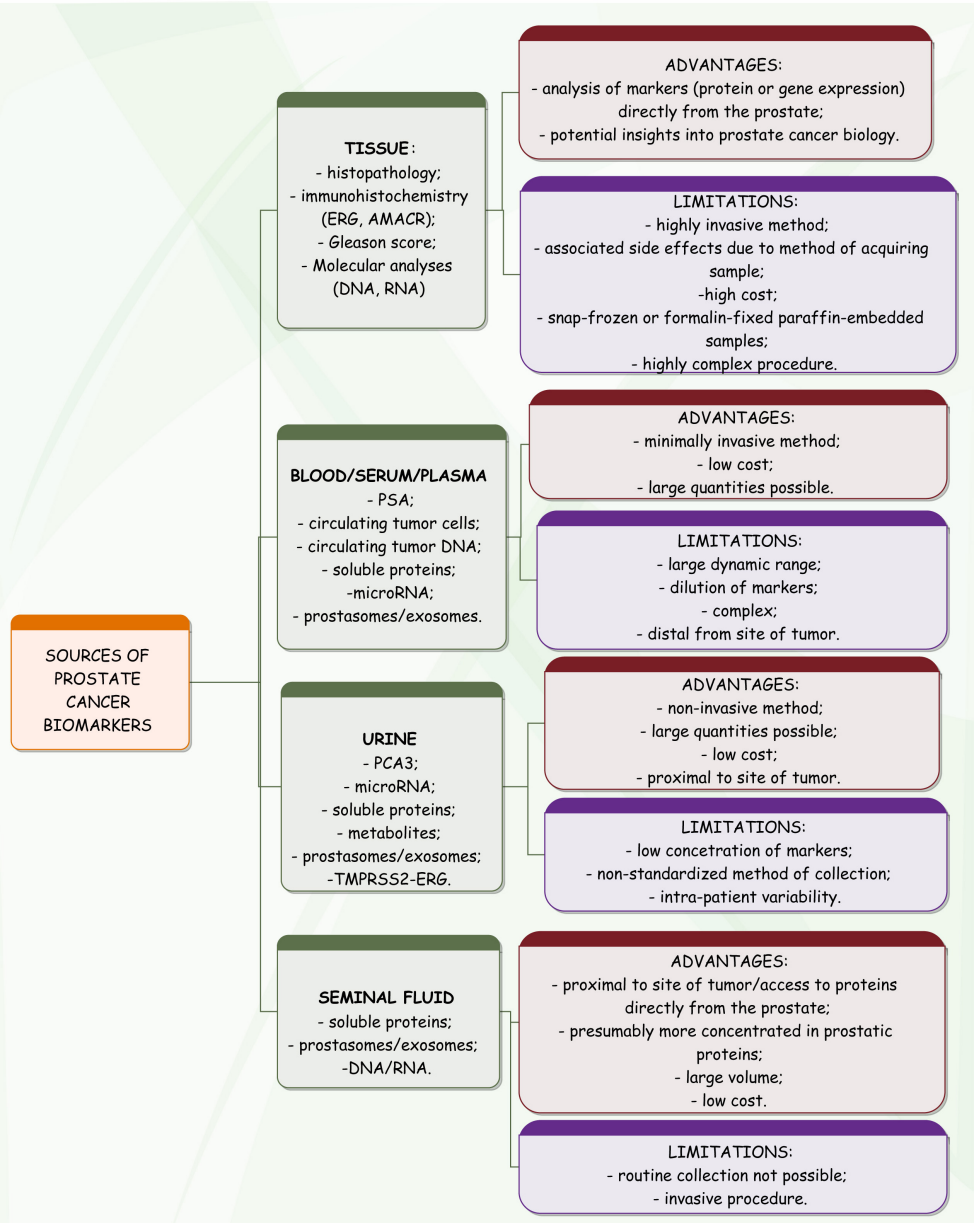
Common sources of biomarkers in prostate cancer

The concept of personalized medicine includes novel protein biomarkers that are expected to improve the early detection, diagnosis and therapy monitoring of PCa [51]. Tissues, biofluids, cell lines and xenograft models are the common sources of biomarker candidates (Figure 2) that require verification of clinical value in independent patient cohorts. Targeted proteomics - based on selected reaction monitoring, or data extraction from data-independent acquisition based digital maps - now represents a promising mass spectrometry alternative to immunochemical methods. To date, it has been successfully used in a high number of studies answering clinical questions on PCa. It plays an important role in functional proteomic experiments that include studying the role of post-translational modifications in cancer progression [52]. The chief aim for PCa biomarker development is to help distinguish indolent from aggressive disease [53]. Almufti et al. propose application of mathematical models for a better prediction of treatment effect, dosing and schedule [54].

### Prostate cancer tissue biomarkers by proteomic analysis

The key to a more effective diagnosis, prognosis, prediction and therapeutic management of PCa could lie in direct analysis of cancer tissue [55]. Prostate tissue has advantage over other biomaterials that in addition of being a rich source of potential PCa biomarkers, offers the possibility to clarify the mechanisms of transformation of a prostate normal cell to a tumor cell and subsequent progression to a metastatic state.

The proteomic analysis of prostate tumor tissue (as a complex mixture of prostate cells, immune and inflammatory cells, blood vessel cells, fibroblasts, nerve cells, endothelial cells, infiltrating lymphocytes, epithelial cells, that cross-talk with each other and collaborate for sustaining tumor growth and proliferation) allows detection of the tumor proteome and/or *in vivo* secretome alterations created by host-tumor cell interactions that may be crucial factors for tumors to undergo progression or regression. The most widely used proteomic technologies are 2DE-MS, MALDI-MS and SELDI-MS, and i-TRAQ, all permitting a qualitative and quantitatively analysis of the proteome variations [4, 55].

A number of comparative proteomic studies have been carried out to find specific diagnostic biomarkers able to distinguish PCa from benign prostatic hyperplasia (BPH) as well as indolent from aggressive cancers. These targets can be grouped in several large categories such as heat shock proteins (e.g. HSP 60, 70), signaling proteins (e.g. PTEN, STAT3) and cytoskeletal proteins (e.g. vinculin, desmin, cytokeratins, even beta-tubulin) (Table 1). Protein biomarkers in tissue.

**Table 1:** Protein biomarkers in tissue

Protein biomarkers	Expression level in PCa tissue	Significance	Proteomic Technologies	Brief results	Refs.
TISSUE BIOMARKERS					
UBE2N (Ubiquitin-conjugating enzyme E2N)	increased	Diagnosis	2-D DIGE, Mass spectrometry	9 proteins were reported for the first time to be modified in PCa	[29]
PSMB6 (Proteasome subunit, beta type, 6)	increased				
PPP1CB (Ser/tre-protein phosphatase PP1β)	decreased				
CPT2 (Carnitinepalmitoyltransferase 2; fatty acid transporter),	increased	Prognostic biomarkers for aggressiveness	Mass spectrometry, IHC	Over 9000 proteins identified in tumor tissue - elevated expression of proteins involved in anabolic processes, ribosomal biogenesis and protein secretion.	[56]
COPA (Coatomer protein complex, subunitα),	increased				
MSK1/2 (Mitogen- and stress-activated protein kinase 1 and 2 protein kinase)	increased				
Pro-NPY	increased
Secernin-1	decreased	Diagnosis and prognosis	2-D DIGE, Mass spectrometry	Secernin-1 and vinculin as potential new tissue biomarkers for PCa; validated using Western blot / immunohistochemistry.	[30]
Vinculin	increased				
NAAA (N-acylethanolamine acid amidase)	decreased	Aggressiveness and metastasis of PCa	SWATH- mass spectrometry; tissue microarray.	220 glycoproteins were associated with PCa aggressiveness and metastasis; two glycoproteins were validated in an independent set of patient tissues by tissue microarray.	[57]
PTK7 (Tyrosine kinase 7)	increased				
TFG (TRK-fused gene)	increased	Diagnosis, prognosis, therapeutic target	LC-MS/MS	TFG expression validated by RT-PCR is associated with higher probability and shorter period of recurrence.	[58]
TTR (Transthyretin)	increased	prognosis; and AAT therapy monitoring	2D-DIGE, MALDI-TOF MS, IHC	Nine proteins were differentially expressed; TTR and CLU - validated by IHC - biomarkers for the prognosis and monitoring the efficacy of androgen ablation therapy.	[59]
CLU( Clusterin)	increased				
MethylcrotonoylCoenzyme A carboxylase 2 (beta) (MCCC2)	increased	tumor progression	2-D DIGE, MS	14 proteins were reported to be differentially expressed between PCa and normal prostate tissue; 3 of them validatated in serum and correlated with 2D-DIGE.	[60]
Tumor necrosis factor receptor-associated Protein 1 (TRAP1)	increased				
Inosine monophosphate dehydrogenase II (IMPDH2)	increased				
HER2/3	increased	PCa stratification, target therapy	Microarray, IHC	Stratification of PCa patients for HER2/3 and PTEN status could identify patients who may respond favorably to MEK inhibition.	[61]
PTEN	decreased				
Periostin (POSTN)	increased	Diagnosis, prognosis and target therapy	iTRAQ, 2D LC-MS/MS	46 proteins were expressed differentially between BPH and PCa and 33 between PCa and BPH with local PIN	[62]
EPLIN (epithelial protein lost in neoplasm)	decreased	Prognosis	cICAT, 2-D LC-MS/MS, Microarray	8 proteins decreased (LIMA1 or EPLIN, S100A4, echinoderm microtubule associated protein like 5, lamin A/C, matrin-3, tubulin-β2C, cytokeratin-18 and −8) and 6 proteins increased (vimentin, keratin II, tropomysin, profilin 1, HSP-β1, and actin-α)	[63]
Androgen receptor isoforms (WT, T877A, and 0CAG)	increased	Diagnosis and prognosis	MS, Gene microarray	2 AR-interaction clusters - 21 and 30 proteins, with unfavourable prognosis outcome	[64]
Eukaryotic initiation factor 4A-III (eIF4A3)	increased	diagnosis and therapeutic strategies	MALDI-TOF-MS/MS, 2-D DIGE	79 different proteins expressed differentially among normal and PCa tissue	[28]
Dimethylargininedimethylaminohydrolase 1 (DDAH1)	increased				
Arginase-2 mitochondrial (ARG2)	increased				
Peroxiredoxins (PRDX3&4)	increased				
Disulfide isomerase (P4HB)	increased	Diagnosis and prognostic monitoring	2-D PAGE, MALDI-ToF MS	22 different proteins differentially expressed in PCa – 5 increased and 5 decreased proteins	[65]
14-3-3 (YWHAG)	increased				
Enoyl CoA-hydrase	increased				
Prohibitin (PHB)	increased				
B-tubulin (TUBB)	increased				
Keratin-II (KRT2)	decreased				
Desmin (DES)	decreased				
HSP71	decreased				
ATP-synthase-β-chain (ATP5B)	decreased				
Creatine kinase-β-chain (CKB)	decreased				
Heat shock protein 60 (HSPD1)	increased	Prognosis	LCM, 2-D DIGE, MALDI-TOF/TOF MS	19 proteins expressed differentially among benign and malignant tumor samples	[66]
Lamin A (LMNA)	increased				
Enhancer of zeste homolog 2 (EZH2)	increased	Prognosis	SID-SRM-MS	EZH2 and AMACR could both mark the presence of an aggressive PCa	[67]
α-methylacyl-CoA recemase (AMACR)	increased				
Cellular retinoic acid-binding protein 2 (CRABP2)	decreased	Novel therapeutic marker	2-D PAGE, MALDI-ToF	Differential protein expression patterns between epithelial and stromal cells isolated from normal, BPH, prostatitis and PCa	[68]
Fatty acid-binding protein, epidermal (FABP5)	increased	Prognosis and diagnosis of aggressive PCa	2-D DIGE, MALDI-TOF/TOF MS PCR, Western blotting, IHC	Out of 58 proteins identified with different expression in the PCa group, 6 proteins were validated as functionally relevant to cancer metastasis.	[69]
Methylcrotonoyl-CoA carboxylase beta chain, mitochondrial (MCCC2)	increased				
Inorganic pyrophosphatase 2 mitochondrial (PPA2)	increased				
Ezrin (EZR)	increased				
SLP2	increased				
SM22	decreased				
Bax, Smac/Diablo phosphorylated Bcl2	increased	Prognosis and stratification for therapy	Reverse phase protein microarray	38 protein signaling - Smac/Diablo and phosphorylated STAT3 (Y705) were found elevated using univariate analysis aggressive PCa	[70]
STAT3 and Smac/Diablo expression	increased				
Prohibitin (PHB)	increased	Diagnosis and target therapy	2-D PAGE, mass spectrometry, IHC	79 different proteins expressed differentially in PCa	[71]
Prostatic acid phosphatase (PAP)	increased				
α-methylacyl CoA racemase (AMACR)	increased	diagnosis, prognosis	iTRAQ , 2-D LC-MS/MS, SRM-MS/MS		30 proteins overexpressed and 35 underexpressed in PCa compared with BPH	[72]
Prostate specific membrane antigen (PSMA)	increased					
Filamin-A FLNA (7–15)	decreased	diagnosis	2-D PAGE, MALDI-TOF-MS/MS	Expression of 3 proteins in PCa tissue validated by immunoblot analyses	[73]
FK506-binding protein 4 (FKBP4)	increased				
Peroxiredoxin-4 (PRDX4)	increased				
Cytokeratins 7, 8 and 18 (KRT7/8/18)	increased	discrimination between low and high GS (Gleason score)	2-D PAGE, mass spectrometry	39 proteins expressed differentially among groups (15 proteins discriminate PCa with low and high aggressiveness; 20 proteins overexpressed and 6 underexpressed in PCa compared with benign samples).	[74]
HSP 60 and 70 (HSPD1, HSPBP1)	increased				
Glutathione S-transferase-π (GSTP1)	increased				
Inorganicpyrophosphatase 2 (PPA2)	increased				
Nucleoside diphosphate kinase 1 (NDPK1)	increased				
Metaxin 2 (MTX2)	increased				
Metalloproteinase inhibitor-1 (TIMP1)	Decreased	Diagnosis	SELDI-TOF, Western blotting, IHC.	Quantitative proteomics was applied; expression pattern was validated by Western blotting and IHC.	[75]
Growth differentiation factor 15 (GDF15)	increased	diagnosis	LCM SELDI-TOF	GDF15 associated with early prostate carcinogenesis	[76]
PCa-24	increased	diagnosis	LCM, SELDI-TOF	Normal and malignant prostate tissues from 17 radical prostatectomy cases analyzed. PCa-24 expression was detected in 94% PCa samples	[77]

Tables 1 and 2 exemplify the variety of biomarkers tested from tissue and serum of patients with PCa. Since absence of a protein is more difficult to evaluate than the presence “positive responses” consisiting in increased expression or combinations of increasedand decreased biomarker levels appear more suitable for diagnosis and prognosis. As such, a group of 52 proteins, among which Filamin A (Decreased), FKB4 and Persiredoxin-4 (Increased) were found by combination of 2D-PAGE and LS/MS in tissue biopsies from PCa and BPH [73]. Differential expression of proteins also discriminates between low and high Gleason scores, as, for instance, the set of KRT 7/8/18, HSPD1, HSPBP1, GST-π, PPA2, NDPK1, MTX2 - found upregulated in high Gleason scores PCa by 2D-PAGE and LC-MS [74], or Bax, Smac/Diablo phosphorylated Bcl2 and STAT3 - used for prognosis and therapy stratification [70] by a combination of microarray and LCMS.

### Prostate cancer biofluids biomarkers by proteomic analysis

Although considered the “gold standard” in PCa diagnosis, biopsy is invasive, correlated with high risk of complications, such as bleeding, sepsis. It also presents 15-20% false negative rate because of ineffective sampling [78]. Consequently, the ideal PCa screening, diagnostics and prognostic biomarkers are yet to be discovered, representing a matter of intense research in body fluids.

### Serum/plasma biomarkers in prostate cancer

A new trend in serum/plasma biomarker search is to go beyond protein, in search of circulating tumor cells or circulating genetic material (DNA, miRNA). However, due to their abundance, proteins still hold the main focus on biomarker research, taking advantage of screening power of new proteomic techniques.

In recent years, due to the development of proteomic and genomic technologies, several prostate biomarkers were analyzed trying to find new tests with higher cancer specificity; these studies were focused mainly on disease diagnostics and less on disease management: prognosis and prediction [79]. Following such studies, novel candidates emerged, such as:caveolins 1 and 2 for disease progression [80], MIC-1 related with tumor progression and low survival [81, 82], complement proteins, to differentiate between malignant and begnin PCA, Pigment epithelium-derived factor (PEDF) as early stage predictor of malignancy [83, 84]. Several other molecules such as, MIF, S100A8/9, Spondin-2, Galectin-3, and Sarcosine were tested as specific prostate cancer biomarkers but with contradictory results or inconsistent data [85].

Proteomic approach, mostly based on mass spectrometry was used to achieve the serum/plasma protein profile of the prostate cancer patients (Table 2).

**Table 2:** Protein biomarkers in serum

Protein biomarkers	Expression level in PCa serum	Significance	Proteomic Technologies	Brief results	Refs.
Caveolin-1 Caveolin-2	increased	therapeutic targets	ELISA, qRT-PCR	- significant correlation between plasma CAV-1 and −2 levels and progression of PC	[80]
Prothrombin	increased	diagnosis	SELDI-ToF-MS; 2-DE; LC-MS/MS	- 20 different protein peaks expressed by SELDI-ToF MS. - 9 unique PCa proteins by 2-DE.	[86]
Complement C4-B (fragment)	increased				
Complement C3 (fragment)	increased				
Zinc-alpha-2-glycoprotein	increased				
Hemopexin	decreased				
Antithrombin-III	decreased				
Pigment epithelium-derived factor	decreased				
Haptoglobin	decreased				
Serum amyloid A-1 protein	decreased				
Pigment epithelium-derived factor (PEDF)	decreased	predictor	2DE, mass spectroscopy	11 altered protein - PEDF involved in prostatic tumorigenesis	[83]
Pigment epithelium-derived factor (PEDF)	decreased	predictor of early stage prostate cancer	2D-DIGE, mass spectroscopy	63 spots differential expression between the Gleason score 5 and 7 cohorts; 13 statistically significant using two independent image analysis packages.	[84]
Zinc-alpha2-glycoprotein (ZAG)	increased				
Complement C4a truncated form (C4a des-Arg)	increased	predicting prostate cancer recurrence	mass spectrometry	30 matched pairs of recurrent and non-recurrent serum samples were randomly selected as a training set for biomarker discovery and model development	[87]
Protein C inhibitor -N-terminal fragment	decreased				

### Prostatic and seminal fluids as sources of proteomic biomarkers for prostate cancer

Prostate is a gland producing a serous secretion (rich in proteins). Because seminal glands open in the prostatic urethra at its initial segment, the expressed prostatic fluid is a combination of both glands. However, clinical collection in the voided urine following prostatic massage is largely devoid of seminal vesicle derived proteins or sperm [88]. The prostatic fluid may contain shed epithelial cells and secreted proteins, recently used for genetic analysis and metabolomic analysis, respectively, in PCa patients.

In order to differentiate between normal and pathologic protein secretion, a detailed investigation of physiologic prostatic fluid was necessary. Mass spectrometry analysis of prostatic secretion in urine revealed over 1000 proteins expressed after a prostatic massage. Out of these, 49 were reported to be specific for prostate [89] and they can be used as comparison between healthy samples and samples form PCa patients. Proteomic components of expressed prostatic fluid harvested from PCa patients have been previously reported [90]. The authors did not, however, validate a panel of several biomarkers, but a bulk of data to be further refined in future studies.

Another use for expressed prostatic fluid is comparison between extra prostatic and organ-confined PCa, which yielded a 34 protein signature to differentiate between the two [91].

Seminal plasma, with its thousands of tissue-specific proteins, also holds great promise for emerging PCa biomarkers [92]. An accurate proteomic analysis was performed on post vasectomy specimens, which, due to ligation of vas deference, are void of testis or epididymis secretions [93]. A recent bioinformatics analysis of published data in the field yielded a set of proteins repeatedly identified, that represent only a fraction of the predicted seminal proteome [94].

Although tumor microenvironment is characterized, amongst other, by increased oxidative stress, measurement of reactive oxygen species in seminal plasma showed no difference between patients with negative or positive prostate biopsy [95].

The possibilities of increased diagnostic accuracy, prediction, and prognostic are expanded with every new data collected from healthy groups and patient cohorts. The main problem to overcome is the massive collection of data - is not feasible to work with hundreds of proteins at a time, but rather a more focused panel is to be desired.

### Prostasomes as a prostate biomarker source

Prostasomes/exosomes are extracellular vesicles secreted by normal and malignant prostate cells but in the case of PCa they appear not only in prostatic fluid but also in peripheral circulation - blood, urine, semen and prostatic fluid [96]. The prostasome levels in plasma of PCa patients can be useful for diagnosis and even prognosis in PCa [97, 98]. Their content has been in-depth analyzed and identified as unique amongst other related exosome proteomes [99]. Multiple prostasomes have been associated with both PCa and elevated Gleason score. They present specific markers (CD46, CD55, CD59), which have a role in the immune system. CD59 levels are more elevated in prostasomes that have been isolated from metastatic prostate cells compared to non-PCa. In addition, they also carry specific molecules, intra and extracellular, that may be specific to PCa and help to discover new PCa biomarkers [100].

## OTHER DIRECTIONS FOR BIOMARKER IDENTIFICATION FOR PROSTATE CANCER DIAGNOSIS AND PROGNOSIS

A step forward in tumor progression study was made by evaluation of circulating tumor cells (CTC). Increased circulating tumor cells in the blood of prostate cancer patients indicated a locally aggressive or metastatic disease and it was associated with poorer overall survival. Moreover, the link between the CTC number and prognosis may be useful for therapy management [101]. The developing of new technologies improved the CTC detection methods which can support the characterization of each tumor, personalized medicine and allows the design of clinical trials testing new compounds against the aggressive cancer cells [102]. Danila et.al identified in CTCs the mRNA of fusion protein TMPRSS2- ERG, but with a limited role as biomarker of sensitivity to abiraterone treatment. This fusion was identified in the biopsies of 30-70% of newly diagnosed PCa patients and the result of the study sustains the use of CTCs as non-invasive method for the PCa diagnosis [103].

Another group of novel serum biomarkers consist of circulating nucleic acids (miRNAs, DNA). Several independent studies identified elevated serum levels of miR-141 and miR-375 to be correlated with metastatic PCa. miR-141 and miR-375 were also correlated with higher Gleason score and positive lymph node status but more studies are required to confirm the potential of these miRNAs as diagnostic and prognostic marker [104].

Another potential non-invasive biomarker for PCa is represented by the presence of methylated GSTP1 DNA in plasma and serum of PCa patients; this epigenetic status was associated with prognosis, advanced AJCC tumor stage, PSA recurrence after surgery and response to chemotherapy. However, hypermethylation of GSTP1 in serum/plasma was identified in about 60% of the patients with confirmed tissue methylation [105].

Though there is a scarce number of studies that assessed whether PCa specific biomarkers are present in peripheral blood samples [106], the results are promising, so more extensive research is required. Even though results seem promising, the main challenge regarding new markers in PCa is their validation in large clinical trials and, consequently, the implementation of these markers into clinical practice.

DNA mutations may be responsible for response to novel molecular treatment, such as PARP inhibitors, as proposed by Mateo et al. [107].

## CONCLUSIONS AND PERSPECTIVES

Proteomic technologies have provided great insight on recent clinical research in biomarker discovery, proving that single-biomarker use is insufficient for an accurate diagnostic and prognostic. The same applies for PCa, in which PSA power of diagnosis and prognosis has been overcome by panels of biomarkers, with better differentiation between indolent and aggressive PCa. Some of these panels have been already developed for current clinical use and are FDA approved. Further development will certainly rely on proteomic platforms to implement high throughput analysis for tissue and biofluids biomarkers. Many pre-clinical and clinical studies are already using proteomics attempting to differentiate between benign and malignancy, but also to understand mechanisms of tumorigenesis and disease progression.

Future ‘must have’ in PCa management will be transition from tumor biopsies to the development of complex fluid-based panels, with superior power over single biomarkers and underlying statistical complexity that should be designed in clinical trials.

Proteomic signature might have a great contribution to personalized approach for PCa diagnosis and treatment outcome prediction. In order to achieve such an ambitious goal, proteomics will need to move from biomarker discovery to rigorous validation process and application of the findings in clinical trials.

Future also holds promise for novel markers such as microRNAs, prostate exosomes or TMPRSS2-ERG fusion product. Prostate-specific exosomes (prostasomes) have a unique protein content specific for PCa and aid in the discovery of new PCa biomarkers. If some of the novel markers have already proven their utility, for the new discover biomarker panels further study will be needed before such markers can be used in standard clinical practice.

In this respect, high-throughput proteomic technologies will hold a greater value in PCa approaches/clinical management, as a basic step to improve diagnostic and prognostic.

## References

[1] Jemal A, Siegel R, Ward E, Hao Y, Xu J, Murray T, Thun MJ (2008). Cancer Statistics, 2008. CA: A Cancer Journal for Clinicians.

[2] Ziaran S, Z Varchulova Novakova, Bohmer D, Danisovic L (2015). Biomarkers for determination prostate cancer: implication for diagnosis and prognosis. Neoplasma.

[3] Tanase C, Albulescu R, Neagu M (2015). Proteomic Approaches for Biomarker Panels in Cancer. Journal of Immunoassay and Immunochemistry.

[4] Pin E, Fredolini C, Petricoin EF (2013). 3rd. The role of proteomics in prostate cancer research: biomarker discovery and validation. Clin Biochem.

[5] Thompson IM, Pauler DK, Goodman PJ, Tangen CM, Lucia MS, Parnes HL, Minasian LM, Ford LG, Lippman SM, Crawford ED, Crowley JJ, Coltman CA (2004). Prevalence of Prostate Cancer among Men with a Prostate-Specific Antigen Level ≤4.0 ng per Milliliter. New England Journal of Medicine.

[6] Dong F, Kattan MW, Steyerberg EW, Jones JS, Stephenson AJ, Schröder FH Klein EA (2008). Validation of Pretreatment Nomograms for Predicting Indolent Prostate Cancer: Efficacy in Contemporary Urological Practice. The Journal of Urology.

[7] Loeb S, Catalona WJ (2013). The Prostate Health Index: a new test for the detection of prostate cancer. Therapeutic Advances in Urology.

[8] Deras IL, Aubin SMJ, Blase A, Day JR, Koo S, Partin AW, Ellis WJ, Marks LS, Fradet Y, Rittenhouse H, Groskopf J (2008). PCA3: A Molecular Urine Assay for Predicting Prostate Biopsy Outcome. The Journal of Urology.

[9] Bollito E, De Luca S, Cicilano M, Passera R, Grande S, Maccagnano C, Cappia S, Milillo A, Montorsi F, Scarpa RM, Papotti M, Randone DF (2012). Prostate cancer gene 3 urine assay cutoff in diagnosis of prostate cancer: a validation study on an Italian patient population undergoing first and repeat biopsy. Anal Quant Cytol Histol.

[10] Ferro M, Bruzzese D, Perdona S, Mazzarella C, Marino A, Sorrentino A, Di Carlo A, Autorino R, Di Lorenzo G, Buonerba C, Altieri V, Mariano A, Macchia V (2012). Predicting prostate biopsy outcome: prostate health index (phi) and prostate cancer antigen 3 (PCA3) are useful biomarkers. Clin Chim Acta.

[11] Auprich M, Bjartell A, Chun FKH, A de la Taille, Freedland SJ, Haese A, Schalken J, Stenzl A, Tombal B, van der Poel H (2011). Contemporary Role of Prostate Cancer Antigen 3 in the Management of Prostate Cancer. European Urology.

[12] Salami SS, Schmidt F, Laxman B, Regan MM, Rickman DS, Scherr D, Bueti G, Siddiqui J, Tomlins SA, Wei JT, Chinnaiyan AM, Rubin MA, Sanda MG (2013). Combining urinary detection of TMPRSS2: ERG and PCA3 with serum PSA to predict diagnosis of prostate cancer. Urologic Oncology: Seminars and Original Investigations.

[13] Stewart GD, Van Neste L, Delvenne P, Delrée P, Delga A, McNeill SA, O’Donnell M, Clark J, Van Criekinge W, Bigley J, Harrison DJ (2013). Clinical Utility of an Epigenetic Assay to Detect Occult Prostate Cancer in Histopathologically Negative Biopsies: Results of the MATLOC Study. The Journal of Urology.

[14] Leyten GHJM, Hessels D, Jannink SA, Smit FP, de Jong H, Cornel EB, de Reijke TM, Vergunst H, Kil P, Knipscheer BC, van Oort IM, Mulders PFA, Hulsbergen-van de Kaa CA (2014). Prospective Multicentre Evaluation of PCA3 and TMPRSS2-ERG Gene Fusions as Diagnostic and Prognostic Urinary Biomarkers for Prostate Cancer. European Urology.

[15] Kami K, Fujimori T, Sato H, Sato M, Yamamoto H, Ohashi Y, Sugiyama N, Ishihama Y, Onozuka H, Ochiai A, Esumi H, Soga T, Tomita M (2012). Metabolomic profiling of lung and prostate tumor tissues by capillary electrophoresis time-of-flight mass spectrometry. Metabolomics.

[16] Partin AW, Van Neste L, Klein EA, Marks LS, Gee JR, Troyer DA, Rieger-Christ K, Jones JS, Magi-Galluzzi C, Mangold LA, Trock BJ, Lance RS, Bigley JW (2014). Clinical validation of an epigenetic assay to predict negative histopathological results in repeat prostate biopsies. J Urol.

[17] Shipitsin M, Small C, Choudhury S, Giladi E, Friedlander S, Nardone J, Hussain S, Hurley AD, Ernst C, Huang YE, Chang H, Nifong TP, Rimm DL (2014). Identification of proteomic biomarkers predicting prostate cancer aggressiveness and lethality despite biopsy-sampling error. British Journal of Cancer.

[18] Gupta A, Roobol MJ, Savage CJ, Peltola M, Pettersson K, Scardino PT, Vickers AJ, Schroder FH, Lilja H (2010). A four-kallikrein panel for the prediction of repeat prostate biopsy: data from the European Randomized Study of Prostate Cancer screening in Rotterdam, Netherlands. Br J Cancer.

[19] Cullen J, Rosner IL, Brand TC, Zhang N, Tsiatis AC, Moncur J, Ali A, Chen Y, Knezevic D, Maddala T, Lawrence HJ, Febbo PG, Srivastava S (2015). A Biopsy-based 17-gene Genomic Prostate Score Predicts Recurrence After Radical Prostatectomy and Adverse Surgical Pathology in a Racially Diverse Population of Men with Clinically Low- and Intermediate-risk Prostate Cancer. Eur Urol.

[20] Knezevic D, Goddard AD, Natraj N, Cherbavaz DB, Clark-Langone KM, Snable J, Watson D, Falzarano SM, Magi-Galluzzi C, Klein EA, Quale C (2013). Analytical validation of the Oncotype DX prostate cancer assay - a clinical RT-PCR assay optimized for prostate needle biopsies. BMC Genomics.

[21] Bishoff JT, Freedland SJ, Gerber L, Tennstedt P, Reid J, Welbourn W, Graefen M, Sangale Z, Tikishvili E, Park J, Younus A, Gutin A, Lanchbury JS (2014). Prognostic utility of the cell cycle progression score generated from biopsy in men treated with prostatectomy. J Urol.

[22] Crawford ED, Scholz MC, Kar AJ, Fegan JE, Haregewoin A, Kaldate RR, Brawer MK (2014). Cell cycle progression score and treatment decisions in prostate cancer: results from an ongoing registry. Current Medical Research and Opinion.

[23] Gaudreau PO, Stagg J, Soulieres D, Saad F (2016). The Present and Future of Biomarkers in Prostate Cancer: Proteomics, Genomics, and Immunology Advancements. Biomark Cancer.

[24] Tanase C, Albulescu R, Neagu M (2016). Proteomic Approaches for Biomarker Panels in Cancer. J Immunoassay Immunochem.

[25] Tanase C, Albulescu R, Codrici E, Popescu ID, Mihai S, Enciu AM, Cruceru ML, Popa AC, Neagu AI, Necula LG, Mambet C, Neagu M (2015). Circulating biomarker panels for targeted therapy in brain tumors. Future Oncol.

[26] Mihai S, Codrici E, Popescu ID, Enciu AM, Rusu E, Zilisteanu D, Albulescu R, Anton G, Tanase C (2016). Proteomic Biomarkers Panel: New Insights in Chronic Kidney Disease. Dis Markers.

[27] Popescu ID, Codrici E, Albulescu L, Mihai S, Enciu AM, Albulescu R, Tanase CP (2014). Potential serum biomarkers for glioblastoma diagnostic assessed by proteomic approaches. Proteome Sci.

[28] Ummanni R, Mundt F, Pospisil H, Venz S, Scharf C, Barett C, Falth M, Kollermann J, Walther R, Schlomm T, Sauter G, Bokemeyer C, Sultmann H (2011). Identification of clinically relevant protein targets in prostate cancer with 2D-DIGE coupled mass spectrometry and systems biology network platform. PLoS One.

[29] Davalieva K, Kostovska IM, Kiprijanovska S, Markoska K, Kubelka-Sabit K, Filipovski V, Stavridis S, Stankov O, Komina S, Petrusevska G, Polenakovic M (2015). Proteomics analysis of malignant and benign prostate tissue by 2D DIGE/MS reveals new insights into proteins involved in prostate cancer. Prostate.

[30] Geisler C, Gaisa NT, Pfister D, Fuessel S, Kristiansen G, Braunschweig T, Gostek S, Beine B, Diehl HC, Jackson AM, Borchers CH, Heidenreich A, Meyer HE (2015). Identification and validation of potential new biomarkers for prostate cancer diagnosis and prognosis using 2D-DIGE and MS. Biomed Res Int.

[31] Geisler C, Gaisa NT, Pfister D, Fuessel S, Kristiansen G, Braunschweig T, Gostek S, Beine B, Diehl HC, Jackson AM, Borchers CH, Heidenreich A, Meyer HE (2015). Identification and Validation of Potential New Biomarkers for Prostate Cancer Diagnosis and Prognosis Using 2D-DIGE and MS. Biomed Res Int.

[32] Popescu DI, Albulescu R, Raducan E, Dinischiotu A, Tanase C (2010). Applications of SELDI-TOF technology in cancer biomarkers discovery. Romanian Biotechnol Lett.

[33] Al-Ruwaili JA, Larkin SE, Zeidan BA, Taylor MG, Adra CN, Aukim-Hastie CL, Townsend PA (2010). Discovery of serum protein biomarkers for prostate cancer progression by proteomic analysis. Cancer Genomics Proteomics.

[34] McLerran D, Grizzle WE, Feng Z, Thompson IM, Bigbee WL, Cazares LH, Chan DW, Dahlgren J, Diaz J, Kagan J, Lin DW, Malik G, Oelschlager D (2008). SELDI-TOF MS whole serum proteomic profiling with IMAC surface does not reliably detect prostate cancer. Clin Chem.

[35] Okamoto A, Yamamoto H, Imai A, Hatakeyama S, Iwabuchi I, Yoneyama T, Hashimoto Y, Koie T, Kamimura N, Mori K, Yamaya K, Ohyama C (2009). Protein profiling of post-prostatic massage urine specimens by surface-enhanced laser desorption/ionization time-of-flight mass spectrometry to discriminate between prostate cancer and benign lesions. Oncol Rep.

[36] Shah P, Wang X, Yang W, S Toghi Eshghi, Sun S, Hoti N, Chen L, Yang S, Pasay J, Rubin A, Zhang H (2015). Integrated Proteomic and Glycoproteomic Analyses of Prostate Cancer Cells Reveal Glycoprotein Alteration in Protein Abundance and Glycosylation. Mol Cell Proteomics.

[37] Adeola HA, Calder B, Soares NC, Kaestner L, Blackburn JM, In Zerbini LF (2016). silico verification and parallel reaction monitoring prevalidation of potential prostate cancer biomarkers. Future Oncol.

[38] Rafalko A, Dai S, Hancock WS, Karger BL, Hincapie M (2012). Development of a Chip/Chip/SRM platform using digital chip isoelectric focusing and LC-Chip mass spectrometry for enrichment and quantitation of low abundance protein biomarkers in human plasma. J Proteome Res.

[39] Cima I, Schiess R, Wild P, Kaelin M, Schuffler P, Lange V, Picotti P, Ossola R, Templeton A, Schubert O, Fuchs T, Leippold T, Wyler S (2011). Cancer genetics-guided discovery of serum biomarker signatures for diagnosis and prognosis of prostate cancer. Proc Natl Acad Sci U S A.

[40] Brazier JS, Smith SA (1989). Evaluation of the Anoxomat: a new technique for anaerobic and microaerophilic clinical bacteriology. J Clin Pathol.

[41] Di Meo A, Pasic MD, Yousef GM (2016). Proteomics and peptidomics: moving toward precision medicine in urological malignancies. Oncotarget.

[42] Boja ES, Fehniger TE, Baker MS, Marko-Varga G, Rodriguez H (2014). Analytical validation considerations of multiplex mass-spectrometry-based proteomic platforms for measuring protein biomarkers. J Proteome Res.

[43] Vialas V, Colome-Calls N, Abian J, Aloria K, Alvarez-Llamas G, Antunez O, Arizmendi JM, Azkargorta M, Barcelo-Batllori S, Barderas MG, Blanco F, Casal JI, Casas V (2016). A multicentric study to evaluate the use of relative retention times in targeted proteomics. J Proteomics.

[44] Rehman I, Evans CA, Glen A, Cross SS, Eaton CL, Down J, Pesce G, Phillips JT, Yen OS, Thalmann GN, Wright PC, Hamdy FC (2012). iTRAQ identification of candidate serum biomarkers associated with metastatic progression of human prostate cancer. PLoS One.

[45] Semmes OJ, Feng Z, Adam BL, Banez LL, Bigbee WL, Campos D, Cazares LH, Chan DW, Grizzle WE, Izbicka E, Kagan J, Malik G, McLerran D (2005). Evaluation of serum protein profiling by surface-enhanced laser desorption/ionization time-of-flight mass spectrometry for the detection of prostate cancer: I. Assessment of platform reproducibility. Clin Chem.

[46] McLerran D, Grizzle WE, Feng Z, Bigbee WL, Banez LL, Cazares LH, Chan DW, Diaz J, Izbicka E, Kagan J, Malehorn DE, Malik G, Oelschlager D (2008). Analytical validation of serum proteomic profiling for diagnosis of prostate cancer: sources of sample bias. Clin Chem.

[47] Grizzle WE, Adam BL, Bigbee WL, Conrads TP, Carroll C, Feng Z, Izbicka E, Jendoubi M, Johnsey D, Kagan J, Leach RJ, McCarthy DB, Semmes OJ (2003). Serum protein expression profiling for cancer detection: validation of a SELDI-based approach for prostate cancer. Dis Markers.

[48] Kerian KS, Jarmusch AK, Pirro V, Koch MO, Masterson TA, Cheng L, Cooks RG (2015). Differentiation of prostate cancer from normal tissue in radical prostatectomy specimens by desorption electrospray ionization and touch spray ionization mass spectrometry. Analyst.

[49] Sawyers CL (2008). The cancer biomarker problem. Nature.

[50] Madu CO, Lu Y (2010). Novel diagnostic biomarkers for prostate cancer. J Cancer.

[51] EC Erno Duda, Popescu Daniela Ionela, Necula Laura (2013). Radu Albulescu. Protein Biomarkers in. Cancers of the Digestive Tract-a Step Towards Personalized Medicine. Current Proteomics.

[52] Pernikarova V, Bouchal P (2015). Targeted proteomics of solid cancers: from quantification of known biomarkers towards reading the digital proteome maps. Expert Rev Proteomics.

[53] Rubin MA (2015). Toward a prostate cancer precision medicine. Urol Oncol.

[54] Almufti R, Wilbaux M, Oza A, Henin E, Freyer G, Tod M, Colomban O, You B (2014). A critical review of the analytical approaches for circulating tumor biomarker kinetics during treatment. Ann Oncol.

[55] Davalieva K, Polenakovic M (2015). Proteomics in diagnosis of prostate cancer. Prilozi.

[56] Iglesias-Gato D, Wikstrom P, Tyanova S, Lavallee C, Thysell E, Carlsson J, Hagglof C, Cox J, Andren O, Stattin P, Egevad L, Widmark A, Bjartell A (2015). The Proteome of Primary Prostate Cancer. Eur Urol.

[57] Liu Y, Chen J, Sethi A, Li QK, Chen L, Collins B, Gillet LC, Wollscheid B, Zhang H, Aebersold R (2014). Glycoproteomic analysis of prostate cancer tissues by SWATH mass spectrometry discovers N-acylethanolamine acid amidase and protein tyrosine kinase 7 as signatures for tumor aggressiveness. Mol Cell Proteomics.

[58] Endoh K, Nishi M, Ishiguro H, Uemura H, Miyagi Y, Aoki I, Hirano H, Kubota Y, Ryo A (2012). Identification of phosphorylated proteins involved in the oncogenesis of prostate cancer via Pin1-proteomic analysis. Prostate.

[59] Wang D, Liang H, Mao X, Liu W, Li M, Qiu S (2012). Changes of transthyretin and clusterin after androgen ablation therapy and correlation with prostate cancer malignancy. Transl Oncol.

[60] Han ZD, Zhang YQ, He HC, Dai QS, Qin GQ, Chen JH, Cai C, Fu X, Bi XC, Zhu JG, Liao DJ, Lu XP, Mo ZY (2012). Identification of novel serological tumor markers for human prostate cancer using integrative transcriptome and proteome analysis. Med Oncol.

[61] Ahmad I, Patel R, Singh LB, Nixon C, Seywright M, Barnetson RJ, Brunton VG, Muller WJ, Edwards J, Sansom OJ, Leung HY (2011). HER2 overcomes PTEN (loss)-induced senescence to cause aggressive prostate cancer. Proc Natl Acad Sci U S A.

[62] Sun C, Song C, Ma Z, Xu K, Zhang Y, Jin H, Tong S, Ding W, Xia G, Ding Q (2011). Periostin identified as a potential biomarker of prostate cancer by iTRAQ-proteomics analysis of prostate biopsy. Proteome Sci.

[63] Zhang S, Wang X, Osunkoya AO, Iqbal S, Wang Y, Chen Z, Muller S, Chen Z, Josson S, Coleman IM, Nelson PS, Wang YA, Wang R (2011). EPLIN downregulation promotes epithelial-mesenchymal transition in prostate cancer cells and correlates with clinical lymph node metastasis. Oncogene.

[64] Paliouras M, Zaman N, Lumbroso R, Kapogeorgakis L, Beitel LK, Wang E, Trifiro M (2011). Dynamic rewiring of the androgen receptor protein interaction network correlates with prostate cancer clinical outcomes. Integr Biol (Camb).

[65] Alaiya AA, Al-Mohanna M, Aslam M, Shinwari Z, Al-Mansouri L, Al-Rodayan M, Al-Eid M, Ahmad I, Hanash K, Tulbah A, A Bin Mahfooz, Adra C (2011). Proteomics-based signature for human benign prostate hyperplasia and prostate adenocarcinoma. Int J Oncol.

[66] Skvortsov S, Schafer G, Stasyk T, Fuchsberger C, Bonn GK, Bartsch G, Klocker H, Huber LA (2011). Proteomics profiling of microdissected low- and high-grade prostate tumors identifies Lamin A as a discriminatory biomarker. J Proteome Res.

[67] Yocum AK, Khan AP, Zhao R, Chinnaiyan AM (2010). Development of selected reaction monitoring-MS methodology to measure peptide biomarkers in prostate cancer. Proteomics.

[68] Khamis ZI, Iczkowski KA, Sahab ZJ, Sang QX (2010). Protein profiling of isolated leukocytes, myofibroblasts, epithelial, Basal, and endothelial cells from normal, hyperplastic, cancerous, and inflammatory human prostate tissues. J Cancer.

[69] Pang J, Liu WP, Liu XP, Li LY, Fang YQ, Sun QP, Liu SJ, Li MT, Su ZL, Gao X (2010). Profiling protein markers associated with lymph node metastasis in prostate cancer by DIGE-based proteomics analysis. J Proteome Res.

[70] Grubb RL, Deng J, Pinto PA, Mohler JL, Chinnaiyan A, Rubin M, Linehan WM, Liotta LA, Petricoin EF, Wulfkuhle JD (2009). Pathway biomarker profiling of localized and metastatic human prostate cancer reveal metastatic and prognostic signatures. J Proteome Res.

[71] Ummanni R, Junker H, Zimmermann U, Venz S, Teller S, Giebel J, Scharf C, Woenckhaus C, Dombrowski F, Walther R (2008). Prohibitin identified by proteomic analysis of prostate biopsies distinguishes hyperplasia and cancer. Cancer Lett.

[72] Garbis SD, Tyritzis SI, Roumeliotis T, Zerefos P, Giannopoulou EG, Vlahou A, Kossida S, Diaz J, Vourekas S, Tamvakopoulos C, Pavlakis K, Sanoudou D, Constantinides CA (2008). Search for potential markers for prostate cancer diagnosis, prognosis and treatment in clinical tissue specimens using amine-specific isobaric tagging (iTRAQ) with two-dimensional liquid chromatography and tandem mass spectrometry. J Proteome Res.

[73] Lin JF, Xu J, Tian HY, Gao X, Chen QX, Gu Q, Xu GJ, Song JD, Zhao FK (2007). Identification of candidate prostate cancer biomarkers in prostate needle biopsy specimens using proteomic analysis. Int J Cancer.

[74] Lexander H, Palmberg C, Hellman U, Auer G, Hellstrom M, Franzen B, Jornvall H, Egevad L (2006). Correlation of protein expression, Gleason score and DNA ploidy in prostate cancer. Proteomics.

[75] Liu AY, Zhang H, Sorensen CM, Diamond DL (2005). Analysis of prostate cancer by proteomics using tissue specimens. J Urol.

[76] Cheung PK, Woolcock B, Adomat H, Sutcliffe M, Bainbridge TC, Jones EC, Webber D, Kinahan T, Sadar M, Gleave ME, Vielkind J (2004). Protein profiling of microdissected prostate tissue links growth differentiation factor 15 to prostate carcinogenesis. Cancer Res.

[77] Zheng Y, Xu Y, Ye B, Lei J, Weinstein MH, O’Leary MP, Richie JP, Mok SC, Liu BC (2003). Prostate carcinoma tissue proteomics for biomarker discovery. Cancer.

[78] Wolters T, van der Kwast TH, Vissers CJ, Bangma CH, Roobol M, Schroder FH, van Leenders GJ (2010). False-negative prostate needle biopsies: frequency, histopathologic features, and follow-up. Am J Surg Pathol.

[79] Prensner JR, Rubin MA, Wei JT, Chinnaiyan AM (2012). Beyond PSA: the next generation of prostate cancer biomarkers. Sci Transl Med.

[80] Sugie S, Mukai S, Yamasaki K, Kamibeppu T, Tsukino H, Kamoto T (2015). Significant Association of Caveolin-1 and Caveolin-2 with Prostate Cancer Progression. Cancer Genomics Proteomics.

[81] Zhao L, Lee BY, Brown DA, Molloy MP, Marx GM, Pavlakis N, Boyer MJ, Stockler MR, Kaplan W, Breit SN, Sutherland RL, Henshall SM, Horvath LG (2009). Identification of candidate biomarkers of therapeutic response to docetaxel by proteomic profiling. Cancer Res.

[82] Brown DA, Lindmark F, Stattin P, Balter K, Adami HO, Zheng SL, Xu J, Isaacs WB, Gronberg H, Breit SN, Wiklund FE (2009). Macrophage inhibitory cytokine 1: a new prognostic marker in prostate cancer. Clin Cancer Res.

[83] Qingyi Z, Lin Y, Junhong W, Jian S, Weizhou H, Long M, Zeyu S, Xiaojian G (2009). Unfavorable prognostic value of human PEDF decreased in high-grade prostatic intraepithelial neoplasia: a differential proteomics approach. Cancer Invest.

[84] Byrne JC, Downes MR, O’Donoghue N, O’Keane C, O’Neill A, Fan Y, Fitzpatrick JM, Dunn M, Watson RW (2009). 2D-DIGE as a strategy to identify serum markers for the progression of prostate cancer. J Proteome Res.

[85] Stephan C, Ralla B, Jung K (2014). Prostate-specific antigen and other serum and urine markers in prostate cancer. Biochim Biophys Acta.

[86] Bergamini S, Bellei E, L Reggiani Bonetti, Monari E, Cuoghi A, Borelli F, Sighinolfi MC, Bianchi G, Ozben T, Tomasi A (2014). Inflammation: an important parameter in the search of prostate cancer biomarkers. Proteome Sci.

[87] Rosenzweig CN, Zhang Z, Sun X, Sokoll LJ, Osborne K, Partin AW, Chan DW (2009). Predicting prostate cancer biochemical recurrence using a panel of serum proteomic biomarkers. J Urol.

[88] Drake RR, White KY, Fuller TW, Igwe E, Clements MA, Nyalwidhe JO, Given RW, Lance RS, Semmes OJ (2009). Clinical collection and protein properties of expressed prostatic secretions as a source for biomarkers of prostatic disease. J Proteomics.

[89] Principe S, Kim Y, Fontana S, Ignatchenko V, Nyalwidhe JO, Lance RS, Troyer DA, Alessandro R, Semmes OJ, Kislinger T, Drake RR, Medin JA (2012). Identification of prostate-enriched proteins by in-depth proteomic analyses of expressed prostatic secretions in urine. J Proteome Res.

[90] Drake RR, Elschenbroich S, Lopez-Perez O, Kim Y, Ignatchenko V, Ignatchenko A, Nyalwidhe JO, Basu G, Wilkins CE, Gjurich B, Lance RS, Semmes OJ, Medin JA (2010). In-depth proteomic analyses of direct expressed prostatic secretions. J Proteome Res.

[91] Kim Y, Jeon J, Mejia S, Yao CQ, Ignatchenko V, Nyalwidhe JO, Gramolini AO, Lance RS, Troyer DA, Drake RR, Boutros PC, Semmes OJ, Kislinger T (2016). Targeted proteomics identifies liquid-biopsy signatures for extracapsular prostate cancer. Nat Commun.

[92] Drabovich AP, Saraon P, Jarvi K, Diamandis EP (2014). Seminal plasma as a diagnostic fluid for male reproductive system disorders. Nat Rev Urol.

[93] Batruch I, Lecker I, Kagedan D, Smith CR, Mullen BJ, Grober E, Lo KC, Diamandis EP, Jarvi KA (2011). Proteomic analysis of seminal plasma from normal volunteers and post-vasectomy patients identifies over 2000 proteins and candidate biomarkers of the urogenital system. J Proteome Res.

[94] Gilany K, Minai-Tehrani A, Savadi-Shiraz E, Rezadoost H, Lakpour N (2015). Exploring the human seminal plasma proteome: an unexplored gold mine of biomarker for male infertility and male reproduction disorder. J Reprod Infertil.

[95] Miriam BM, Carlos AG, AG MJ, Joan PL (2015). Diagnosis of prostate cancer by analyzing oxidative stress in human seminal plasma: developing unsophisticated tools for noninvasive prostate cancer diagnosis. Eur J Cancer Prev.

[96] Overbye A, Skotland T, Koehler CJ, Thiede B, Seierstad T, Berge V, Sandvig K, Llorente A (2015). Identification of prostate cancer biomarkers in urinary exosomes. Oncotarget.

[97] Tavoosidana G, Ronquist G, Darmanis S, Yan J, Carlsson L, Wu D, Conze T, Ek P, Semjonow A, Eltze E, Larsson A, Landegren UD, Kamali-Moghaddam M (2011). Multiple recognition assay reveals prostasomes as promising plasma biomarkers for prostate cancer. Proc Natl Acad Sci U S A.

[98] Lance RS, Drake RR, Troyer DA (2011). Multiple recognition assay reveals prostasomes as promising plasma biomarkers for prostate cancer. Expert Rev Anticancer Ther.

[99] Principe S, Jones EE, Kim Y, Sinha A, Nyalwidhe JO, Brooks J, Semmes OJ, Troyer DA, Lance RS, Kislinger T, Drake RR (2013). In-depth proteomic analyses of exosomes isolated from expressed prostatic secretions in urine. Proteomics.

[100] Velonas VM, Woo HH, dos Remedios CG, Assinder SJ (2013). Current status of biomarkers for prostate cancer. Int J Mol Sci.

[101] Delacruz A (2012). Using circulating tumor cells as a prognostic indicator in metastatic castration-resistant prostate cancer. Clin J Oncol Nurs.

[102] Kolostova K, Broul M, Schraml J, Cegan M, Matkowski R, Fiutowski M, Bobek V (2014). Circulating tumor cells in localized prostate cancer: isolation, cultivation in vitro and relationship to T-stage and Gleason score. Anticancer Res.

[103] Danila DC, Anand A, Sung CC, Heller G, Leversha MA, Cao L, Lilja H, Molina A, Sawyers CL, Fleisher M, Scher HI (2011). TMPRSS2-ERG status in circulating tumor cells as a predictive biomarker of sensitivity in castration-resistant prostate cancer patients treated with abiraterone acetate. Eur Urol.

[104] Kuner R, Brase JC, Sultmann H, Wuttig D (2013). microRNA biomarkers in body fluids of prostate cancer patients. Methods.

[105] Ellinger J, Muller SC, Dietrich D (2015). Epigenetic biomarkers in the blood of patients with urological malignancies. Expert Rev Mol Diagn.

[106] Dijkstra S, Mulders PF, Schalken JA (2014). Clinical use of novel urine and blood based prostate cancer biomarkers: a review. Clin Biochem.

[107] Mateo J, Carreira S, Sandhu S, Miranda S, Mossop H, Perez-Lopez R, D Nava Rodrigues, Robinson D, Omlin A, Tunariu N, Boysen G, Porta N, Flohr P (2015). DNA-Repair Defects and Olaparib in Metastatic Prostate Cancer. N Engl J Med.

